# Differential expression of αVβ3 and αVβ6 integrins in prostate cancer progression

**DOI:** 10.1371/journal.pone.0244985

**Published:** 2021-01-22

**Authors:** Fabio Quaglia, Shiv Ram Krishn, Yanqing Wang, David W. Goodrich, Peter McCue, Andrew V. Kossenkov, Amy C. Mandigo, Karen E. Knudsen, Paul H. Weinreb, Eva Corey, William K. Kelly, Lucia R. Languino

**Affiliations:** 1 Prostate Cancer Discovery and Development Program, Thomas Jefferson University, Philadelphia, PA, United States of America; 2 Department of Cancer Biology, Thomas Jefferson University, Philadelphia, PA, United States of America; 3 Department of Pharmacology & Therapeutics, Roswell Park Comprehensive Cancer Center, Buffalo, NY, United States of America; 4 Department of Pathology, Thomas Jefferson University, Philadelphia, PA, United States of America; 5 Center for Systems and Computational Biology, Wistar Institute, Philadelphia, PA, United States of America; 6 Biogen Inc., Cambridge, MA, United States of America; 7 Department of Urology, University of Washington, Seattle, Washington, United States of America; 8 Department of Medical Oncology, Thomas Jefferson University, Philadelphia, PA, United States of America; University of Minnesota Twin Cities, UNITED STATES

## Abstract

Neuroendocrine prostate cancer (NEPrCa) arises *de novo* or after accumulation of genomic alterations in pre-existing adenocarcinoma tumors in response to androgen deprivation therapies. We have provided evidence that small extracellular vesicles released by PrCa cells and containing the αVβ3 integrin promote neuroendocrine differentiation of PrCa *in vivo* and *in vitro*. Here, we examined αVβ3 integrin expression in three murine models carrying a deletion of *PTEN* (SKO), *PTEN* and *RB1* (DKO), or *PTEN*, *RB1* and *TRP53* (TKO) genes in the prostatic epithelium; of these three models, the DKO and TKO tumors develop NEPrCa with a gene signature comparable to those of human NEPrCa. Immunostaining analysis of SKO, DKO and TKO tumors shows that αVβ3 integrin expression is increased in DKO and TKO primary tumors and metastatic lesions, but absent in SKO primary tumors. On the other hand, SKO tumors show higher levels of a different αV integrin, αVβ6, as compared to DKO and TKO tumors. These results are confirmed by RNA-sequencing analysis. Moreover, TRAMP mice, which carry NEPrCa and adenocarcinoma of the prostate, also have increased levels of αVβ3 in their NEPrCa primary tumors. In contrast, the αVβ6 integrin is only detectable in the adenocarcinoma areas. Finally, analysis of 42 LuCaP patient-derived xenografts and primary adenocarcinoma samples shows a positive correlation between αVβ3, but not αVβ6, and the neuronal marker synaptophysin; it also demonstrates that αVβ3 is absent in prostatic adenocarcinomas. In summary, we demonstrate that αVβ3 integrin is upregulated in NEPrCa primary and metastatic lesions; in contrast, the αVβ6 integrin is confined to adenocarcinoma of the prostate. Our findings suggest that the αVβ3 integrin, but not αVβ6, may promote a shift in lineage plasticity towards a NE phenotype and might serve as an informative biomarker for the early detection of NE differentiation in prostate cancer.

## Introduction

Integrins are transmembrane adhesion receptors that are deregulated during cancer progression [1, 2]. Among others, αVβ6, αVβ3, α6β1, and α6β4 integrins are overexpressed in in prostate cancer (PrCa) [3–6]; our group recently demonstrated that small extracellular vesicles released from PrCa cells and containing the αVβ3 integrin induce neuroendocrine differentiation (NED) *in vitro* and *in vivo* [7]. In contrast, the α5 and α7 integrin subunits have been reported to be downregulated in PrCa [8].

The αVβ3 integrin, also known as the vitronectin receptor, is composed of two subunits, αV and β3. It can bind a wide range of extracellular matrix components through its RGD motif (Arg-Gly-Asp) [9] and promotes invasion and adhesion of cancer cells to extracellular matrix proteins [2, 10, 11]. This RGD-integrin binding is also known to facilitate cell adhesion, virus entry, and infection by many human viruses [12], including metapneumovirus [13] and coxsackievirus [14]. According to a recent study, the interaction between the RGD motif in the spike protein of the SARS-Cov-2 virus (responsible for COVID-19) and integrins may promote the entry of the virus into the host cells [15]. The αVβ3 integrin itself is involved in a variety of processes, including angiogenesis and tumor metastasis [16]. While present at very low levels in normal prostate tissues, it is highly expressed in PrCa cells and in metastasis [7, 10, 17]. Given its widespread distribution in PrCa, αVβ3 has been explored as a therapeutic target in some studies [18, 19].

Dysregulated expression of the αVβ6 integrin, another RGD binding integrin, has been associated with poor outcomes in different types of cancer [20]. Previous studies from our group showed that αVβ6 integrin is upregulated in PrCa and PrCa bone metastases [21, 22].

Neuroendocrine PrCa (NEPrCa), a subtype of PrCa that typically develops from subsets of castrate-resistant PrCa (CRPrCa) cells, is highly aggressive and usually metastasizes [23]. NEPrCa tumors may develop *de novo* or through the acquisition of alterations in pre-existing epithelial tumors in response to therapies as outlined in the recent National Cancer Institute workshop “Perspective on Lineage Plasticity and AR-independent PrCa” [24]. *De novo* NEPrCa appears to result from lineage reprogramming of mature differentiated cells that do not express androgen receptor (AR) or prostate-specific antigen (PSA) but instead express neuron-specific proteins, such as aurora kinase A (AURKA), synaptophysin (SYP), and neuron-specific enolase (NSE) [25–27]. These aberrations promote pro-tumorigenic pathways independently from those activated by the AR [28]. Treatment-emergent NEPrCa has similar characteristics but, at variance, it acquires expression of the AR [29]. From a clinical perspective, NEPrCa quickly develops resistance to chemotherapy and is associated with a life expectancy of less than one year [25, 30].

Here we show, for the first time, that αVβ3 integrin expression is increased in NEPrCa, but absent in prostatic adenocarcinomas (ADPrCa). Our immunohistochemical analysis of PrCa samples reveals differential expression of the αVβ3 and αVβ6 integrins. We find that the αVβ3 integrin is highly expressed in metastases from NEPrCa patients while αVβ6 integrin is mostly expressed in ADPrCa lacking neuroendocrine features. We also show that αVβ3 expression is increased in a murine model that lacks the *PTEN*, *RB1*, and *TRP53* genes and develops NEPrCa resembling its human counterpart. Loss of *PTEN* and *RB1*, with intact *TRP53*, also causes increased expression of αVβ3 integrin, although to a lower extent. Moreover, we report increased αVβ3 integrin expression in the tumors of TRAMP (Transgenic Adenocarcinoma of the Mouse Prostate) mice that develop NEPrCa together with castrate-sensitive ADPrCa. We confirmed these results by screening of 42 LuCaP patient-derived xenografts (PDXs) [31, 32]. Our analysis uncovers a positive correlation between αVβ3 integrin and SYP but not between αVβ6 and this NE marker. Our study provides novel insights into the identification of new pathways that might promote lineage plasticity among PrCa subtypes for which there is no established therapeutic approach. The differential expression of these two lineage-restricted integrins might also serve as a useful biomarker to predict neuroendocrine differentiation and facilitate patient stratification in PrCa.

## Materials and methods

### Cell lines

PrCa C4-2B and LNCaP cell culture conditions have been previously described [10, 33].

### Antibodies

**Fig 1:**
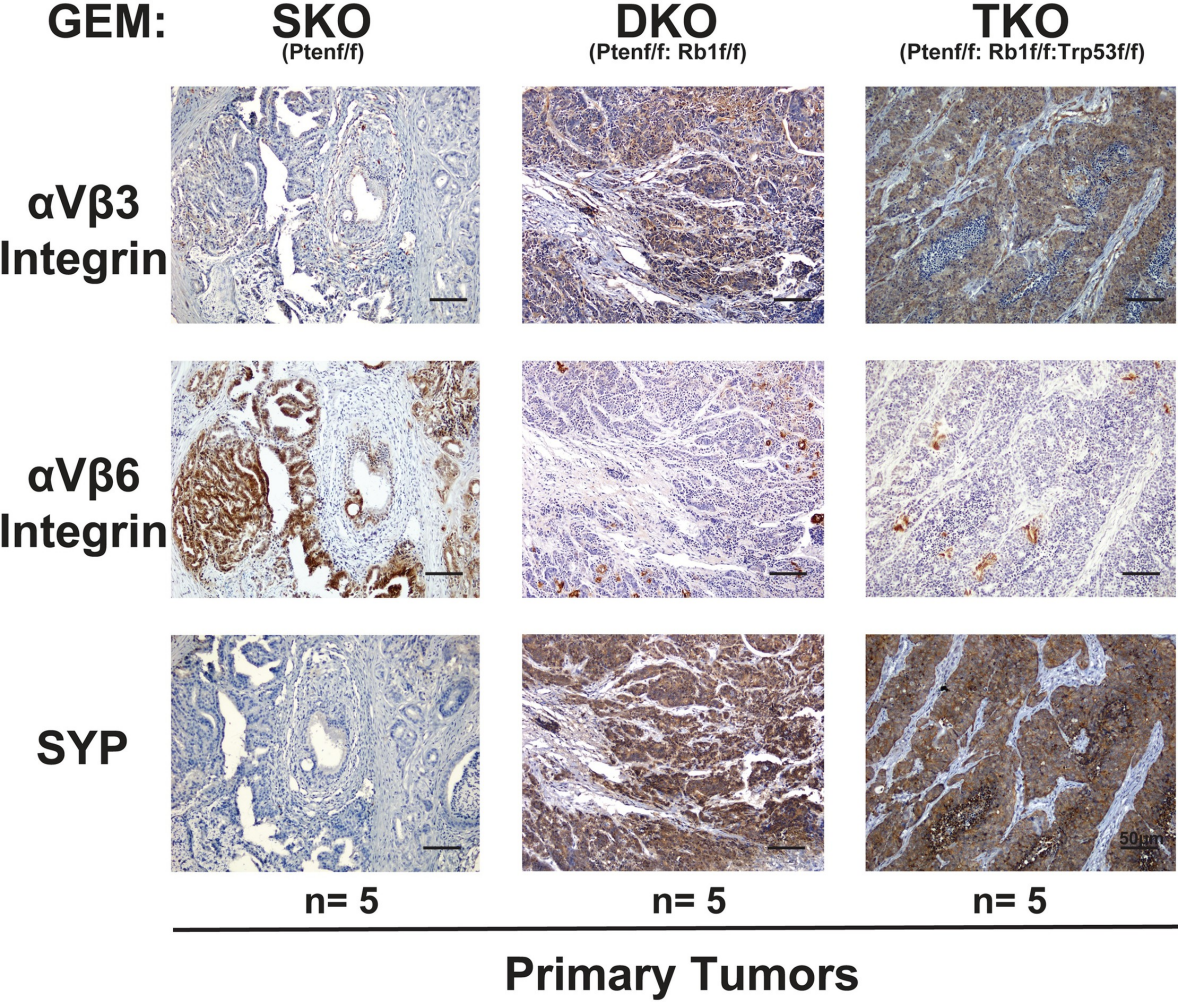
αVβ3 integrin is selectively upregulated in the primary tumors of mice carrying neuroendocrine prostate cancer. Immunostaining of the αVβ3 integrin (top panels), αVβ6 integrin (middle panels), and SYP (bottom panels) in prostate tumors from murine models with genetic knockdown of PTEN (SKO; n = 5), PTEN and RB1 (DKO; n = 5), and PTEN, RB1, and TRP53 (TKO; n = 5) in the prostatic epithelium. The bar at the bottom right corner of each panel represents 50 μm. First column: SKO; second column: DKO; third column: TKO.

**Fig 2:**
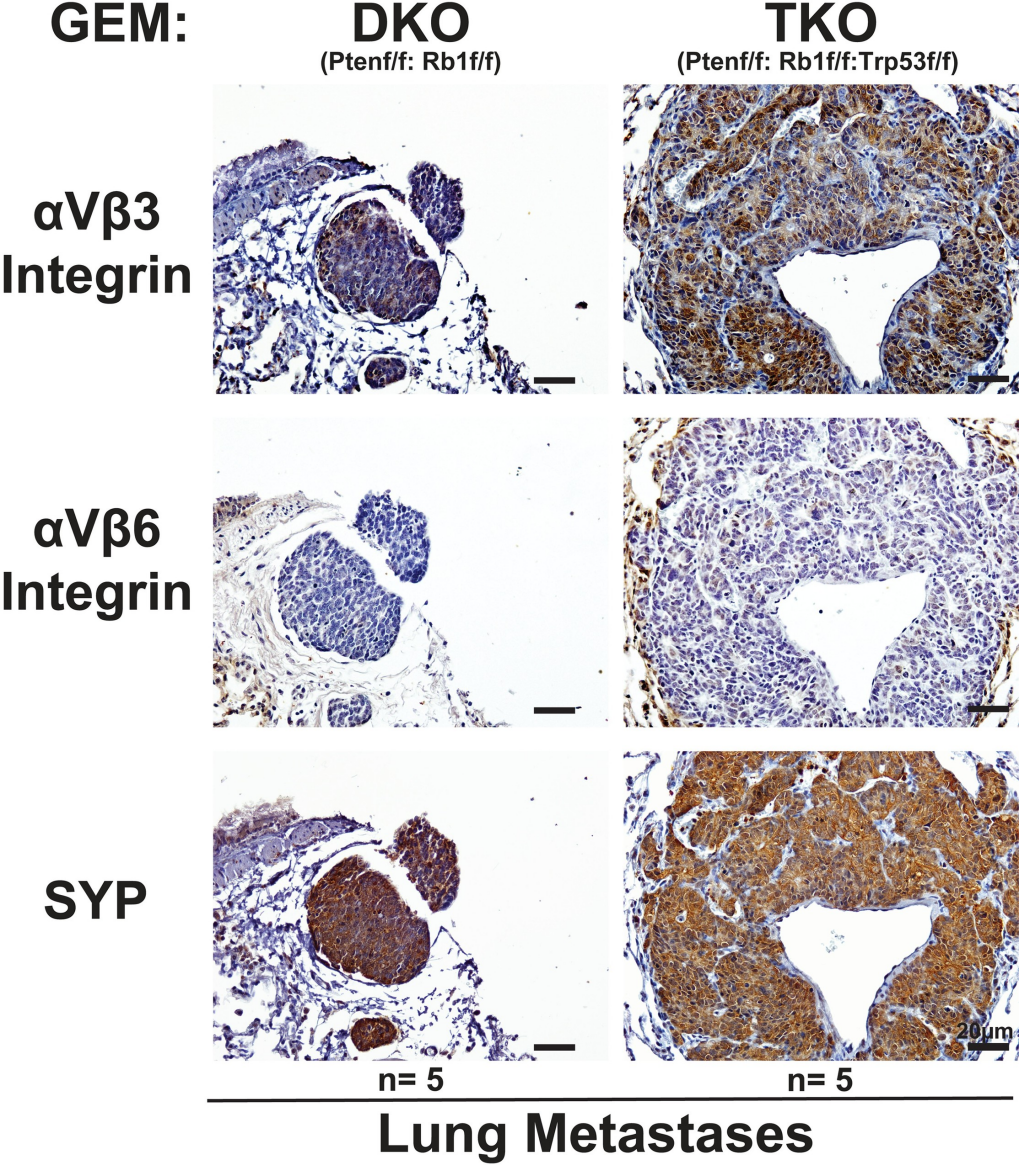
αVβ3 integrin is selectively upregulated in lung metastases of mice carrying neuroendocrine prostate cancer. Immunostaining of the αVβ3 integrin (top panels), αVβ6 integrin (middle panels), and SYP (bottom panels) in the lung metastases from murine models with genetic knockdown of PTEN and RB1 (DKO; n = 5) and PTEN, RB1, and TRP53 (TKO; n = 5) in the prostatic epithelium. The bar at the bottom right corner of each panel represents 20 μm. First column: DKO; second column: TKO.

Immunohistochemistry (IHC) analysis used two different rabbit monoclonal antibodies (Abs) against β3 integrin subunit: one from Cell Signaling (13166S; Figs 1 and 2) and another from AbCam (Ab75872; Fig 4). Moreover, a rabbit polyclonal Ab against SYP (Invitrogen, PA1-1043) and a rabbit polyclonal Ab against chromogranin A (CgA, Invitrogen, 18–0094) were used. For the β6 integrin subunit, a mouse monoclonal Ab against the β6 integrin subunit (6.2A1) [34] was used for immunostaining of human samples, and a human/mouse chimeric Ab against the β6 integrin subunit (ch2A1) [35] was used for SKO, DKO, and TKO murine samples. Immunoblotting analysis used rabbit monoclonal Ab against β3 integrin subunit (Cell Signaling, 13166S), rabbit polyclonal Abs against TSG101 (Abcam, ab30871), actin (Sigma, A2066), and a mouse monoclonal Ab against the β6 integrin subunit (6.2A1).

### Generation of mice carrying prostate-specific TRP53 and RB1 gene deletions

Mice of genotype PB-Cre4 *PTEN*^*loxP/loxP*^, PB-Cre4 *PTEN*^*loxP/loxP*^*RB1*^*loxP/loxP*^, or PB-Cre4 *PTEN*^*loxP/loxP*^ *RB1*^*loxP/loxP*^*TRP53*^*loxP/loxP*^ were generated as previously described [36, 37]. Briefly, mice carrying different combinations of the *PTEN*^*loxP*^, *RB1*^*loxP*^, and *TRP53*^*loxP*^ alleles were interbred, with the ARR2PB-Cre transgene from the PB-Cre4 line always carried through males. Mice used in this analysis are on a C57BL/6 and 129SVJ mixed genetic backgrounds. Mice were backcrossed to the C57BL/6 strain for at least 5 generations. Genotypes were designated as SKO (single *PTEN* knock-out), DKO (double *PTEN*:*RB1* knock-out), and TKO (triple *PTEN*:*RB1*:*TRP53* knock-out). Non-recombinant littermates were used as a control. The mice were euthanized using CO_2_ and cervical dislocation when the tumor length was approximately 2 cm. All of these mice were maintained following guidelines of the Institutional Animal Care and Use Committee (IACUC), and were bred and kept at Roswell Park Comprehensive Cancer Center (Buffalo, NY, USA).

### TRAMP (Transgenic Adenocarcinoma of the Mouse Prostate) mice

Male TRAMP mice were generated as described previously [38]. Twenty-four male TRAMP mice were used. No female mice were analyzed in this study. The mice were euthanized using CO_2_ and cervical dislocation when the tumor volume was approximately 10,000 mm^3^. Care of animals was in compliance with standards established by the Office of Laboratory Animal Welfare, NIH, Department of Health and Human Services. All mice were maintained following recommendations of the IACUC. Experimental protocols were approved by IACUC.

### PDX establishment

The acquisition of PrCa patient tissues and their use to establish PDX models have been described [32]. The vast majority of implanted tissues was from metastatic foci obtained at tissue acquisition necropsy in a manner which limited warm ischemic time as much as possible (aiming for 4–8 hours after death). A few samples of primary PrCa were obtained from surgical procedures. Harvested tumor tissues were evaluated by pathologists, and viable tumor tissue was macro-dissected to minimize content of stroma, fat, and necrotic tissue. Tumor fragments were implanted subcutaneously in 6- to 8-week-old intact male athymic Nu/Nu (NU‐*Foxn1nu)* or CB‐17 severe combined immunodeficient (SCID, CB17/Icr‐*Prkdcscid*/IcrCrl) mice (Charles River Laboratory). Tumor samples were harvested from later passages (>3) and frozen or embedded in paraffin for characterization. LuCaP PDXs are maintained by constant passaging in SCID mice. The levels of SYP in the LuCaP PDX were assessed by IHC analysis.

### Immunohistochemistry (IHC)

IHC was performed on tissue sections from SKO (n = 5), DKO (n = 5), and TKO (n = 5) prostate tumors and lung metastases, from TRAMP murine primary tumors, and on LuCaP PDX TMA containing 42 PDX models. Of the 24 TRAMP mice analyzed, 13 exhibited a NE phenotype, 11 presented adenocarcinoma lesions, and 5 displayed both characteristics. The tissue sections were baked at 60°C for 1 hour, followed by deparaffinization with xylene (3 min × 2), and rehydration through a graded ethanol series (100%, 90%, 70%, 50%, 30% for 3 min each) followed by deionized water (3 min × 2). The sections were incubated with 3% H_2_O_2_ solution for quenching endogenous peroxidase activity, followed by heat-induced antigen retrieval for the β3 integrin subunit, SYP or chromogranin (CgA) that was performed in citrate buffer (10 mM sodium citrate, 0.05% Tween 20, pH 6.0) at 95°C for 15 min. For β6 integrin subunit immunostaining, antigen retrieval was performed using pepsin (0.5% in 5 mM HCl) digestion for 15 min at 37°C. Sections were washed once with deionized water for 5 min, followed by a phosphate buffer saline (PBS) wash for 5 min, and blocked with 5% goat serum in PBST (PBS, 0.1% Tween20) for 2 hours. The tissue sections were incubated overnight at 4°C with Abs against β3 integrin subunit (1:25), β6 integrin subunit (2 μg/ml), CgA (1 μg/ml), SYP (5 μg/ml), or the respective IgG isotype, which was used as negative control. The following day, the tissue sections were washed with PBST (5 min × 2), followed by PBS (5 min), and incubated with secondary Abs (biotinylated goat anti-rabbit IgG in PBST for β3 integrin, SYP, or CgA, and biotinylated goat anti-human or horse anti-mouse IgG for β6 integrin, 10 μg/ml in PBST) for 30 min at room temperature. The unbound secondary Ab was washed with PBST (5 min × 2), followed by PBS (5 min). The tissue sections were incubated with streptavidin horseradish peroxidase (SAP, 5 μg/ml in PBS) for 30 min at room temperature and the unbound SAP was washed with PBST (5 min × 2), followed by PBS (5 min). The chromogenic reaction product was developed by adding substrate chromogen 3,3′-diaminobenzidine solution (DAB substrate kit). The DAB reaction was stopped by rinsing the tissue sections in deionized water. The sections were counterstained with Harris hematoxylin, dehydrated in a graded ethanol series (30%, 50%, 70%, 90%, 100% for 5 min each) followed by xylene (5 min × 2), dried, and finally mounted with Permount (Vector Laboratories).

### LuCaP TMA immunohistochemical assessment and statistical analysis

LuCaP PDX TMA immunostaining was scored by multiplying each staining intensity level (“0” for no stain, “1” for faint stain, and “2” for definitive stain) by the percentage of cells at each staining level. The multiplicands provided a final score for each sample (score range was 0 to 200). The score for each LuCaP core was the average of the scores of each triplicate. Relative detection levels of SYP were provided by Dr. Corey and defined as 0 (-), 1 (+), 2 (++), and 3 (+++). The normalization was performed by assigning to the higher score for each immunostaining (αVβ3, αVβ6, and SYP) a value of 100. Correlation analysis between the integrin scores and the expression levels of SYP and its significance was performed using Spearman correlation (Matlab v.R2016a).

### RNA-sequencing (RNA-seq)

RNA-seq was performed as previously reported in [39] and publicly available on GEO Expression Omnibus (accession number: GSE90891). Briefly, RNA-seq was performed on SKO (n = 4), DKO (n = 5), and TKO (n = 4) prostate tumors and on normal prostate (n = 4) by the Roswell Park Cancer Institute Genomics shared resource. Sequencing libraries were prepared with the TruSeq Stranded Total RNA kit (Illumina Inc) from 1 μg total RNA following manufacturer’s instructions. After ribosomal RNA depletion, RNA was purified, fragmented, and primed for cDNA synthesis. Fragmented RNA was reverse transcribed into first-strand cDNA using random primers. AMPure XP beads were used to separate the cDNA from the second-strand reaction mix resulting in blunt-ended cDNA. A single ‘A’ nucleotide was then added to the 3’ ends of the blunt fragments. Multiple indexing adapters, containing a single ‘T’ nucleotide on the 3’ end of the adapter, were ligated to the ends of the cDNA to prepare them for hybridization onto a flow cell. Libraries were purified and validated for the appropriate size on a 2100 Bioanalyzer High Sensitivity DNA chip (Agilent Technologies, Inc.). The DNA library was quantitated using KAPA Biosystems qPCR kit and normalized to 2 nM prior to pooling. Libraries were pooled in an equimolar fashion and diluted to 10 pM. Library pools were clustered and run on a HiSeq2500 rapid mode sequencer according to the manufacturer's recommended protocol (Illumina Inc.).

Raw sequencing reads passing the Illumina RTA quality filter were pre-processed using FASTQC for sequencing base quality control. Reads were mapped to the mouse reference genome (mm9) and RefSeq annotation database using Tophat. A second round of quality control using RSeQC was applied to mapped bam files to identify potential RNA-seq library preparation problems. The number of reads aligning to each gene was calculated using HTSeq, and for each gene, the corresponding RPKM value was calculated.

For differential gene expression analysis, RNA-seq counts were processed to remove genes lacking expression in more than 80% of samples. Scale normalization was done using the Limma package in R. After Voom transformation, data from primary SKO, DKO, and TKO tumors were compared to generate differentially expressed gene lists with P < 0.05 and logFC > 1.5.

### Human subject inclusion criteria

Three metastatic ADPrCa tissue samples (Gleason Score GS 9 [n = 1] and GS 10 [n = 2]) were obtained from the Department of Pathology at Thomas Jefferson University (Philadelphia, PA). Additionally, four human malignant ADPrCa tissue samples (GS 7 [n = 3] and GS 10 [n = 1]) were obtained from the Cooperative Human Tissue Network (CHTN) Western Division at Vanderbilt University Medical Center, TN, or Mid-Atlantic Division at University of Virginia, VA. The CHTN is funded by the National Cancer Institute and other investigators may have received specimens from the same subjects. All specimens were de-identified and discarded in accordance with IRB-approved protocols.

### siRNA transfection and immunoblotting analysis

Downregulation of AR was accomplished using siRNA SMARTPool (Dharmacon, L-003400-00-0005) and non-targeting siRNA as a control (Dharmacon, D-001810-10-05). Transfection of siRNA and immunoblotting analysis were performed as previously described [21].

## Results

### The αVβ3 integrin is selectively upregulated in NEPrCa murine models

In a recent study, we have shown that the αVβ3 integrin is found in small extracellular vesicles released by cancer cells and that small extracellular vesicles containing αVβ3 have a unique ability to promote NED of PrCa *in vivo* [7]. Based on these findings, we hypothesized that elevated expression levels of αVβ3 might correlate with NED in PrCa. We tested this hypothesis by analyzing the levels of αVβ3 and αVβ6 integrins in primary tumors, as well as lung metastatic lesions, from NEPrCa mice carrying *PTEN*, *RB1*, and *TRP53* triple conditional knock-outs in the prostatic epithelium (PBCre4 *PTEN*^*loxP/loxP*^ *RB1*^*loxP/loxP*^*TRP53*^*loxP/loxP*^, TKO). This model has been reported to develop NEPrCa similar to its human counterpart [39]. We compared the TKO model to a double knock-out model lacking *PTEN* and *RB1* in the prostate (PBCre4 *PTEN*^*loxP/loxP*^ *RB1*^*loxP/loxP*^, DKO). In addition, we analyzed a PTEN single conditional knock-out mouse model (PBCre4 *PTEN*^*loxP/loxP*^, SKO) whose gene expression signature has been shown to be comparable to human ADPrCa [39]. The immunostaining analysis reveals high levels of the αVβ3 integrin (Figs 1 and 2, top panels) which correlate with SYP expression (Figs 1 and 2, bottom panels) in the prostate tumors (Fig 1) and lung metastatic lesions (Fig 2) of DKO and TKO mice (n = 5 for each group). The results are consistent in all samples except for one of the DKO samples which does not exhibit detectable αVβ3 integrin expression. In the tumors from the SKO mice, the αVβ3 integrin is not detectable (Fig 1, top panels), whereas the αVβ6 integrin is highly expressed in SKO prostate tumor samples (Fig 1, middle panels), and is low with some patchy positivity in the DKO and TKO primary tumors (Fig 1, middle panels). Consistent with these results, lung metastatic lesions from DKO and TKO mice show some patchy positivity for the αVβ6 integrin (Fig 2, middle panels) but at a considerably lower level than for αVβ3. We did not observe any metastases in SKO mice.

Consistent with the immunostaining results, RNA sequencing analysis of the publicly available datasets on Geo Expression Omnibus (GSE90891, [39]) reveals higher levels of the β3 integrin subunit (*ITGB3*) expression in DKO and TKO tumors compared to SKO samples. Moreover, *ITGB3* mRNA is upregulated in SKO compared to normal prostate (wild type, WT; Table 1), although our immunostaining analysis does not detect the αVβ3 integrin in the SKO samples analyzed (Fig 1). These results indicate that, although the *ITGB3* mRNA is present in SKO tumors, the mRNA is likely to be unstable. In addition, the levels of the αV integrin subunit (*ITGAV*) and β6 integrin subunit (*ITGB6*) are lower in DKO and TKO tumors compared to SKO, although noticeably higher in all three knock-out genotypes compared to normal prostate (WT) samples (Table 1).

**Table 1:** RNA sequencing analysis shows increased expression of ITGB3 mRNA in DKO and TKO tumors.

	WT (n = 4)	SKO (n = 4)	DKO (n = 5)	TKO (n = 4)
*ITGB3*	23.8	1145.5	3536.4	3018
*ITGAV*	764.3	22121.3	3262.6	4857.3
*ITGB6*	22.8	3652.5	2113.8	1033
*PARP1*	580.8	4434.3	15946.2	10862.5
*POU3F4*	0	0	86.6	75.5

Normalized read counts for the β3 integrin subunit (ITGB3), αV integrin subunit (ITGAV), β6 integrin subunit (ITGB6), PARP1, and BRN4 (POU3F4) RNA levels in normal prostate (WT), as well as in SKO, DKO, and TKO prostate tumor samples.

NEPrCa expresses elevated levels of PARP1 which is a nuclear enzyme involved in DNA repair, DNA replication, inflammation, and chromosome organization [40, 41]. Consistent with these previous publications, *PARP1* expression is upregulated in DKO and TKO tumors (Table 1). Although *PARP1* mRNA is also upregulated in SKO samples compared to the WT control, the levels of *PARP1* mRNA are not as elevated as in DKO and TKO tumors (Table 1). In addition, another gene involved in NED (BRN4 [*POU3F4*]) [42] is upregulated in DKO and TKO samples but not in SKO (Table 1). These results demonstrate that high expression of *ITGB3* and of genes implicated in NED co-occur in DKO and TKO tumors.

**Fig 3:**
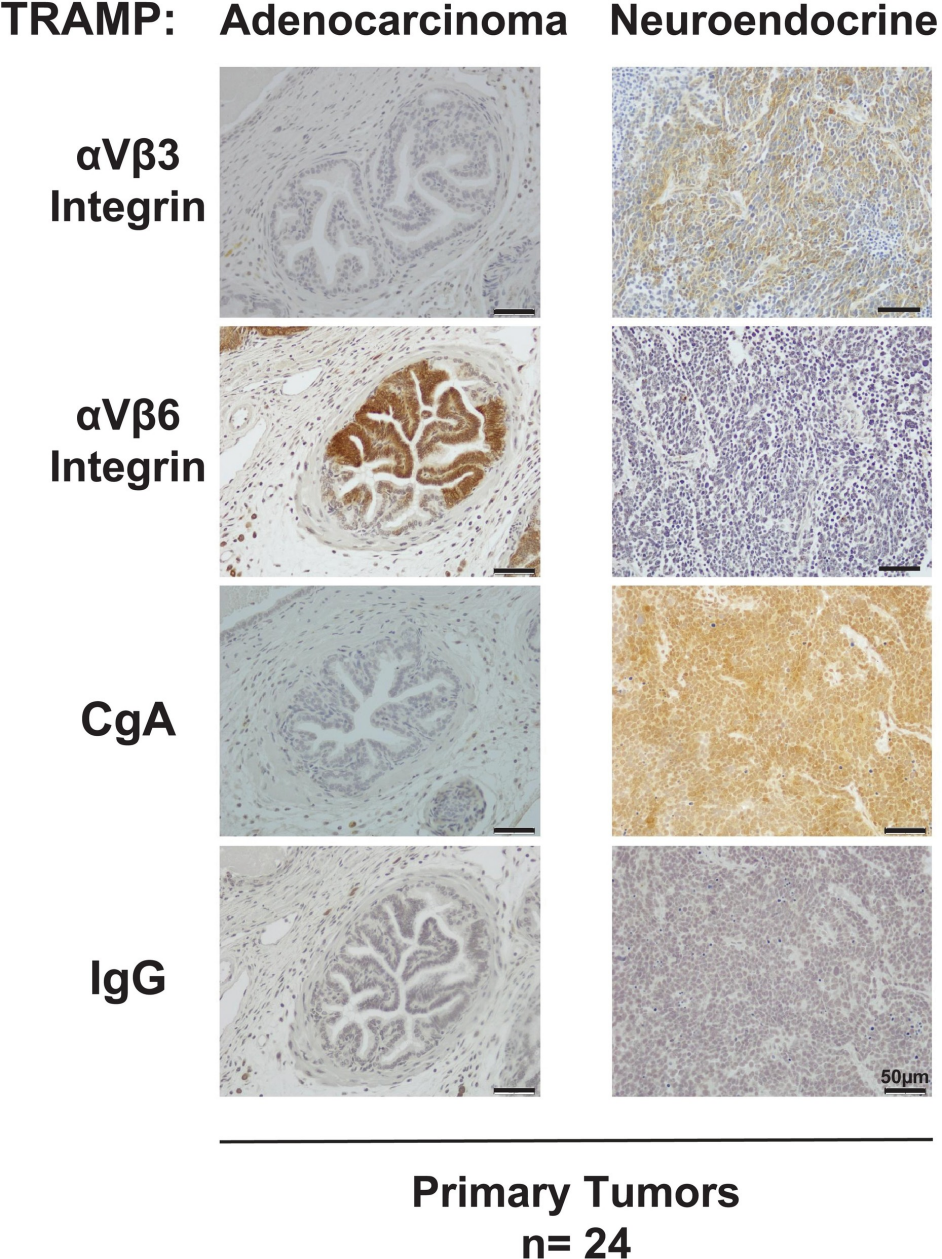
Selective upregulation of αVβ3 integrin in the TRAMP (Transgenic Adenocarcinoma of the Mouse Prostate) mice. IHC staining of αVβ3 (first row), αVβ6 (second row), and chromogranin A (CgA, third row) of prostate tumors from TRAMP mice (n = 24). Of the 24 samples analyzed, 13 show only a NE phenotype, 11 show only ADPrCa lesions, and 5 show both characteristics. IgG was used as negative control (last row). The bar at the bottom right corner of each panel represents 50 μm. Left column, ADPrCa; right column, NEPrCa.

We also performed immunohistochemical analysis of tumor samples from TRAMP mice to assess the levels of αVβ3 and αVβ6 integrin expression in their tumors. This mouse model, which is known to have RB and p53 inactivated, develops NEPrCa together with ADPrCa [43, 44]. Our immunostaining shows that the NE marker chromogranin A (CgA) co-occurs with the αVβ3 integrin in 10 of the 13 TRAMP NE tumor samples analyzed (Fig 3). The αVβ6 integrin, however, is not detected in the NE tumors from the TRAMP model (Fig 3). In contrast, the αVβ6 integrin is detected exclusively in the ADPrCa, NE-negative areas of the TRAMP tumor samples (Fig 3). Our results, from the DKO and TKO NE mouse genetic models as well as the TRAMP mice, taken together, clearly demonstrate a consistent correlation between the high expression of αVβ3 integrin and NEPrCa occurrence. Conversely, ADPrCa tumors are consistently associated with expression of the alternative αVβ6 integrin subtype.

### Expression of αVβ3 integrin and synaptophysin correlates in patient-derived xenografts

**Fig 4:**
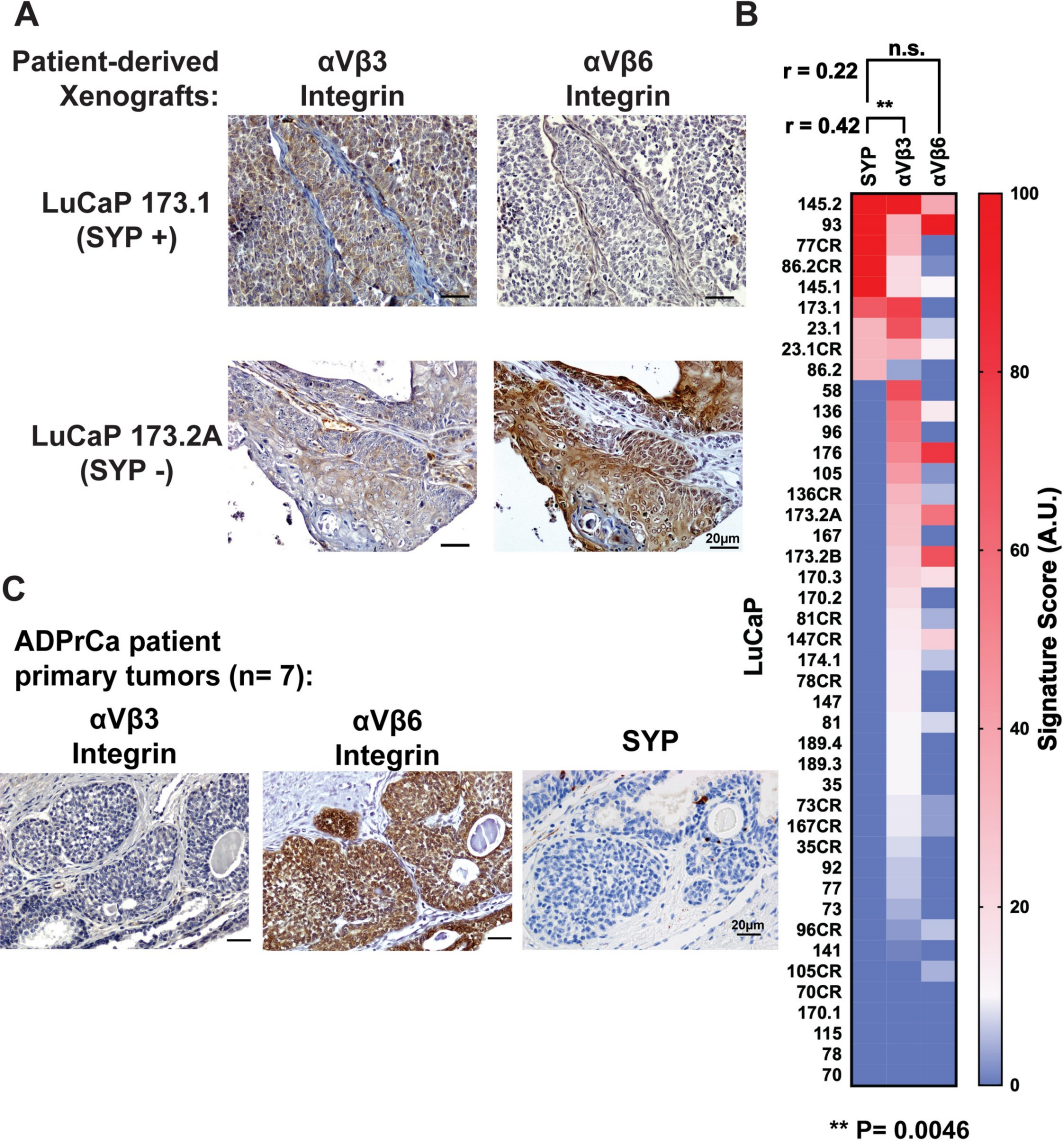
Increased expression of the αVβ3 integrin, but not αVβ6, correlates with the neuroendocrine marker SYP in LuCaP PDXs and human prostate tumor samples. Immunohistochemical analysis of 42 LuCaP PDX models. (A) representative IHC staining for αVβ3 (left) or αVβ6 (right) integrin of SYP positive (top row) or SYP-negative (bottom row) LuCaP PDX models is shown. The bar at the bottom right corner of each panel represents 20 μm. (B) Heat map of the signature score for SYP, αVβ3 or αVβ6 integrin of each LuCaP is shown. Raw data are reported in the S1 Table. (C) Immunostaining analysis of αVβ3 and αVβ6 integrins and SYP primary tumors from ADPrCa patients. The bar at the bottom right corner of each panel represents 20 μm.

To confirm these results in human specimens, we conducted an immunohistochemical analysis of 42 LuCaP PDXs [31, 32]. These PDX models were generated by implanting primary PrCa or metastatic lesion tumor fragments from PrCa patients into immunocompromised mice [32], and the resulting PDX models were subsequently characterized for their expression of NE markers [31]. We assessed the presence of αVβ3 or αVβ6 integrin using immunohistochemical analysis and scored the immunostaining intensity of each LuCaP core in the tumor micro-array (TMA) using the scoring system described in the Materials and Methods section. We observe a positive correlation between the αVβ3 integrin and the NE marker SYP (Fig 4A and 4B, r = 0.42; P = 0.0046). In contrast, the αVβ6 integrin shows no correlation with SYP (Fig 4A and 4B, r = 0.22; P = 0.1622), confirming the results described above obtained for mouse tumor samples.

We further validated the results obtained using the LuCaP PDX TMA by screening PrCa samples from the Department of Pathology at Thomas Jefferson University and the Cooperative Human Tissue Network. Of the 7 ADPrCa primary tumors none expresses αVβ3 (Fig 4C). On the other hand, as previously reported [21], most of the ADPrCa express αVβ6 which was used as positive control. These findings suggest a differential expression of these two αV integrins during PrCa progression, whereby the αVβ3 integrin is specifically expressed in NEPrCa samples, and in contrast, the αVβ6 integrin is specifically expressed in ADPrCa samples lacking NE characteristics.

### Loss of androgen receptor signaling does not result in upregulation of αVβ3 or αVβ6 integrin expression in PrCa cell lines

**Fig 5:**
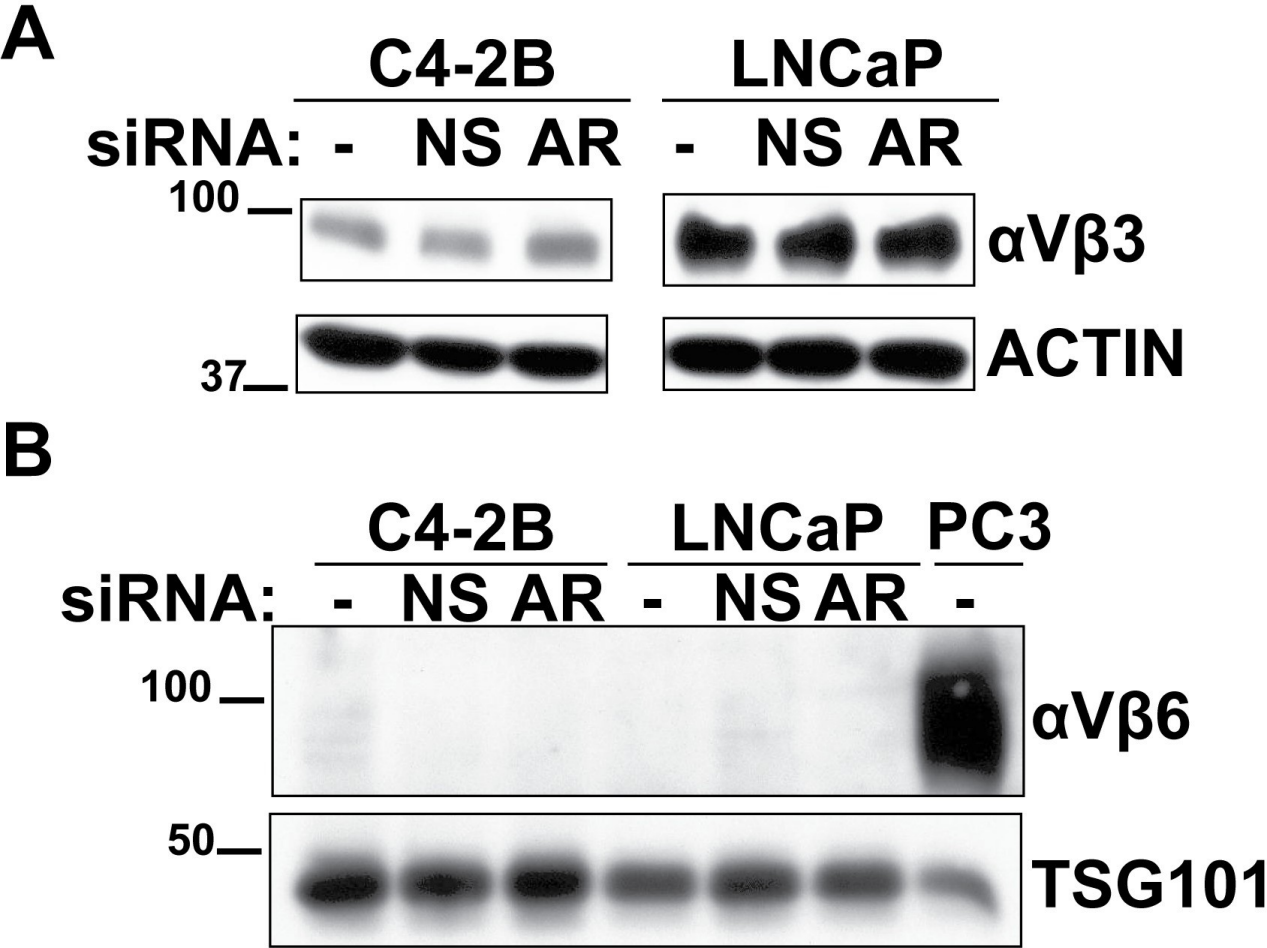
Downregulation of androgen receptor does not increase αVβ3 or αVβ6 integrin expression. Immunoblotting analysis of C4-2B and LNCaP cell lysates after AR downregulation by siRNA to AR. (A) Expression levels of αVβ3 integrin in C4-2B and LNCaP cells after AR downregulation. Immunoblotting was performed under reducing conditions. (B) Expression levels of αVβ6 integrin in C4-2B and LNCaP cells after AR downregulation. Immunoblotting was performed under non-reducing conditions. Actin or TSG101 serves as loading controls. NS, non-silencing.

NEPrCa is characterized by the activation of pro-tumorigenic pathways independently from the AR signaling [28]. We hypothesized that loss of AR signaling might induce upregulation of the αVβ3 integrin in LNCaP and C4-2B, two AR positive PrCa cell lines. To test our hypothesis, we downregulated AR expression in LNCaP and C4-2B cells using siRNA. Our results show that downregulation of AR in C4-2B or LNCaP cells does not upregulate αVβ3 (Fig 5A) or αVβ6 integrin (Fig 5B) expression. Thus, it is possible that other factors in the tumor microenvironment contribute to the regulation of αVβ3 integrin and αVβ6 integrin expression after AR signaling loss.

## Discussion

Our results demonstrate that increased expression of the αVβ3 integrin correlates with the occurrence of NE markers in human patients’ samples and murine models. In contrast, the αVβ6 integrin is expressed in human and murine ADPrCa, suggesting that the αVβ3, but not αVβ6, integrin might serve as a suitable biomarker to characterize NED in the context of PrCa.

Here, we show that these two integrins are differentially expressed in ADPrCa and NE cancers. Specifically, expression of the αVβ3 integrin in primary prostate tumors and metastatic lesions of mice carrying deletions of the *PTEN* (SKO), *RB1* and *PTEN* (DKO) or *RB1*, *PTEN*, and *TP53* (TKO) inversely correlates with αVβ6. Expression of the αVβ3 integrin in primary prostate tumors of mice carrying deletions of the *PTEN* (SKO) is undetectable, while it is significantly increased in DKO or TKO tumors and metastatic lesions. This indicates that *RB1* loss, and consequent activation of transcription factors of the E2F family [45–47], is sufficient to induce αVβ3 expression in these models. This integrin expression persists in TKO tumors which, in contrast to DKO tumors exhibiting both SYP and AR expression, develop homogenous AR-negative NEPrCa, similar to its human counterpart [39]. It remains to be investigated whether downregulation of αVβ6 and gain of the αVβ3 integrin occur in CRPrCa since RB1 is known to influence integrin expression [48, 49], and its loss occurs frequently in human CRPrCa [50, 51].

A factor that may influence the processing of the αVβ3 integrin, is the expression of the αV subunit which is required for the heterodimeric complex. The RNA analysis summarized here (Table 1) indicates that the levels of the αV integrin subunit (*ITGAV*) become limiting and that β3 acts in a dominant fashion over the β6 integrin subunit.

We also detect high αVβ3 integrin expression in the NE areas of primary tumors from TRAMP mice that develop NEPrCa together with ADPrCa. In contrast, we detect the related αVβ6 integrin in the ADPrCa areas of the TRAMP tumors. Our findings underline the specificity of the αVβ3 integrin in NEPrCa, nominating this integrin as a potential biomarker for patient stratification in PrCa treatment. Our future studies will benefit from the use of mice carrying deletion of the αVβ3 integrin crossed with the DKO, TKO, or TRAMP mice, in order to shed new light on the mechanism of action of the αVβ3 integrin in NEPrCa development and/or metastatic progression.

**Fig 6:**
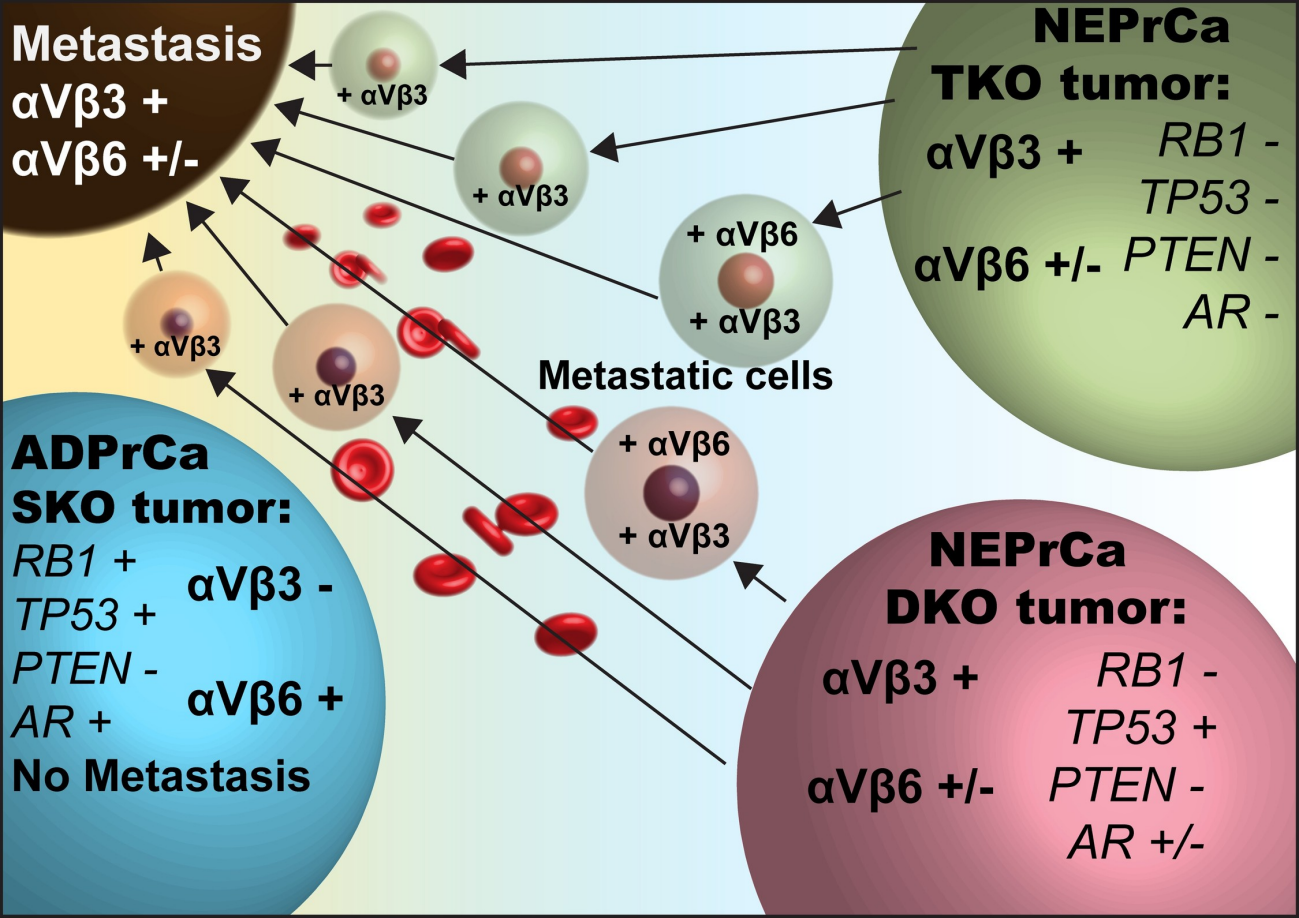
Schematic representation of the findings described in this study. SKO (PB-Cre4 PTEN^loxP/loxP^) cancer cells do not metastasize and express low levels of αVβ3 integrin and high levels of αVβ6 integrin. On the other hand, DKO and TKO tumors (PB-Cre4 PTEN^loxP/loxP^ RB1^loxP/loxP^ and PB-Cre4 PTEN^loxP/loxP^ RB1^loxP/loxP^ TRP53^loxP/loxP^ respectively) express high levels of αVβ3 integrin and low levels of αVβ6 integrin. These αVβ3 positive tumors acquire metastatic behavior and expression of NE markers.

Multiple strategies have been developed to target the αVβ3 integrin due to its role in tumor angiogenesis and tumor growth [16]. For example, LM609, an inhibitory antibody against the αVβ3 integrin, reduced angiogenesis and tumor growth in a SCID mouse/human chimeric model for breast cancer [52]. Its humanized counterpart JC-7U IgG1 has been reported to inhibit tumor growth in a Kaposi sarcoma mouse model and was also able to inhibit, in part, the binding of human immunodeficiency virus (HIV-1) Tat protein to αVβ3 integrin, which is necessary to stimulate Kaposi sarcoma growth [16, 53]. Previous studies also reported the ability of the αVβ3 integrin to support metastasis in PrCa [54] as well as other cancers [55–58]. Likewise, the expression of the αVβ3 integrin conceivably facilitates the metastatic behavior of NEPrCa. In support of this idea, our SKO mouse model (PB-Cre4 *PTEN*^*loxP/loxP*^*)* does not metastasize and expresses low levels of αVβ3 integrin, whereas DKO and TKO, the two NE models that acquire αVβ3 integrin expression as a consequence of additional *RB1* knock-out, develop metastases in the lungs [39]. We can speculate that upon RB1 loss, downregulation of αVβ6 and gain of the αVβ3 integrin are required in the primary tumors in the early stages of NED to confer upon NEPrCa the ability to metastasize in different sites (Fig 6). Upon metastasizing, the αVβ3 integrin expression is sustained as shown here and as previously described [7] in NEPrCa bone metastasis, indicating additional pro-survival functions provided by this integrin.

Whether one or more of the many pathways activated by the αVβ3 integrin is involved in NED remains to be established. For example, the expression of the αVβ3 integrin reportedly stimulates cell migration by activation of the phosphatidylinositol 3-kinase (PI3K)/AKT pathway [59]. Other studies have demonstrated that AKT1 is involved in stabilizing N-MYC [60, 61], one of the main promoters of NED in PrCa [62]. Since pAKT is not detectable in TKO prostate tissue [39], we speculate that pAKT activated by αVβ3 primes the cells to stabilize N-MYC but is not required for long-lasting NED. The RNA-seq analysis presented here highlights potential downstream effectors of αVβ3. For example, αVβ3 integrin might be able to induce NED in PrCa by upregulating Trop2 expression, which is known to induce NEPrCa by upregulation of PARP1 [40]. Underlining the importance of targeting this pathway to prevent or delay the most aggressive forms of PrCa, the U.S. Food and Drug Administration has recently approved olaparib, a PARP1 inhibitor [63], for the treatment of metastatic CRPrCa. However, there are as yet no reports on the safety or efficacy of olaparib for the treatment of NEPrCa.

Our previous study demonstrates that the dysregulated expression of the αVβ3 integrin in small extracellular vesicles released by PrCa cells promotes a shift in lineage plasticity towards a NE lineage [7]. Moreover, although our group has reported that the αVβ6 integrin, in small extracellular vesicles released by cancer cells, induces M2 polarization in recipient monocytes [64] and stimulates angiogenesis in endothelial cells during cancer progression [65], is absent in NEPrCa. Here we show that the αVβ3 integrin is upregulated in tumor samples from patients affected by NEPrCa and in corresponding NE murine models. Moreover, our findings demonstrate that conversely, the expression of the αVβ6 integrin is upregulated in ADPrCa samples from humans and mice. It is therefore reasonable to speculate that monitoring the expression of these two integrins during PrCa progression will help to predict the potential for NED in PrCa patients. Moreover, based on our emerging findings that NE metastatic lesions express relatively high levels of the αVβ3 integrin, targeted therapies directed against this integrin might prove to be effective in preventing or delaying plasticity and metastasis in NEPrCa [56].

## Supporting information

S1 Table Raw data of the signature score used to generate the heatmap in Fig 4. (TIF)

S1 Raw images (PDF)
