# Supplementary figures and images for: Differential expression of αVβ3 and αVβ6 integrins in prostate cancer progression
Source: PLoS One. 2021 Jan 22;16(1):e0244985. doi: 10.1371/journal.pone.0244985 (PMC7822502; doi:10.1371/journal.pone.0244985)

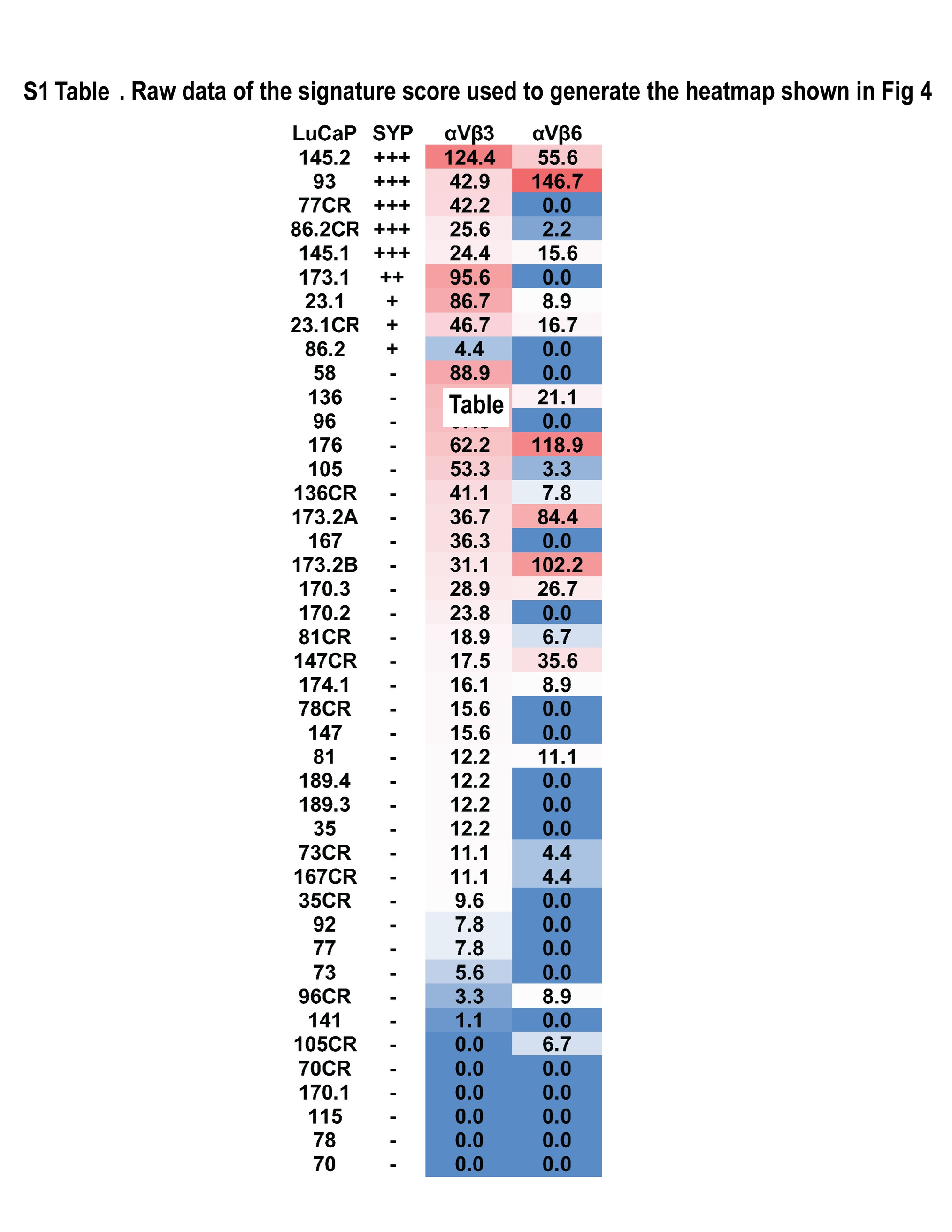

Supplement: S1 Table — (TIF) [file pone.0244985.s001.tif]
