# Supplementary material for: Differential expression of αVβ3 and αVβ6 integrins in prostate cancer progression
Source: PLoS One. 2021 Jan 22;16(1):e0244985. doi: 10.1371/journal.pone.0244985 (PMC7822502; doi:10.1371/journal.pone.0244985)
Supplement: S1 Raw images — (PDF) [file pone.0244985.s002.pdf]

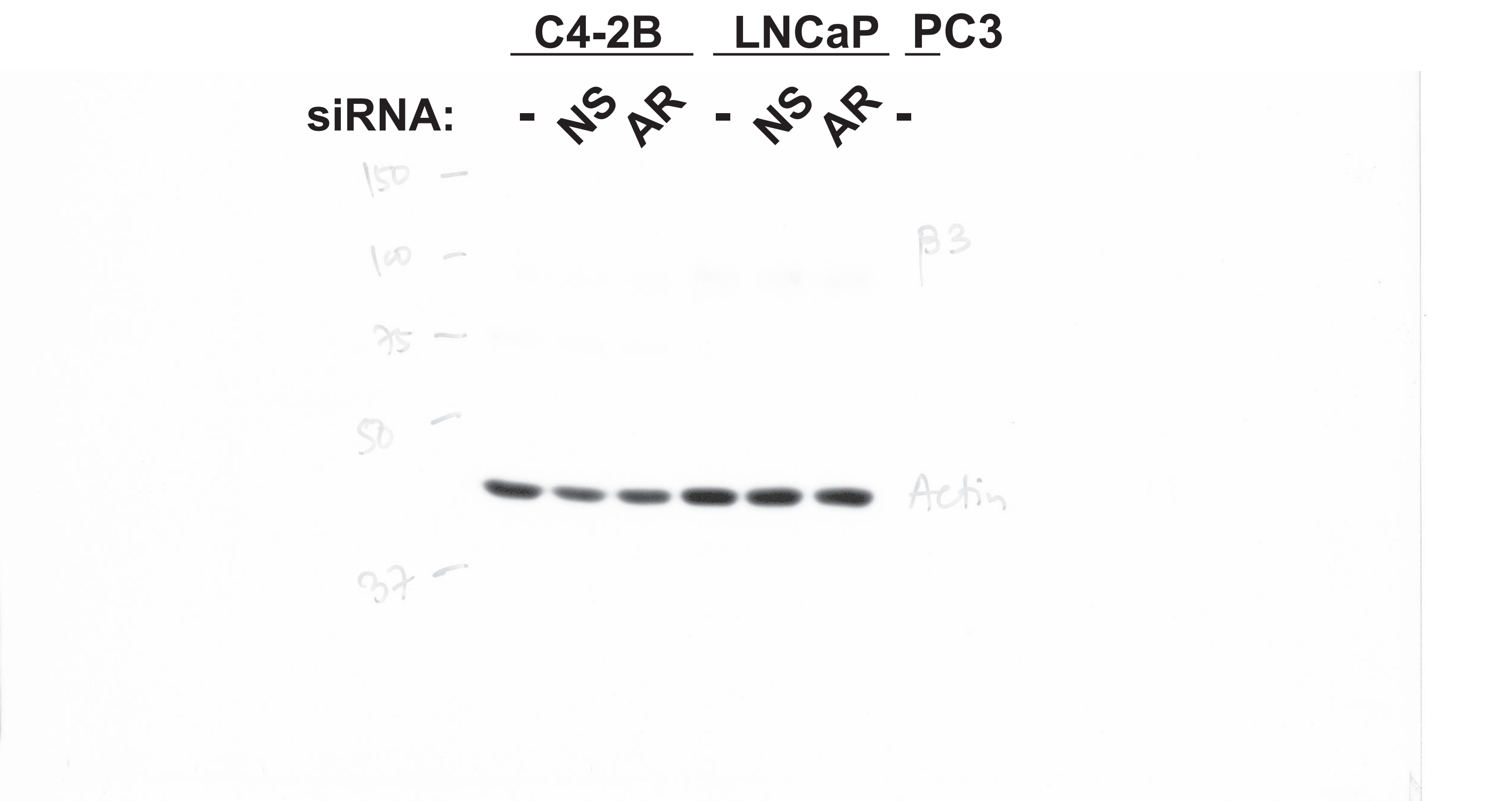

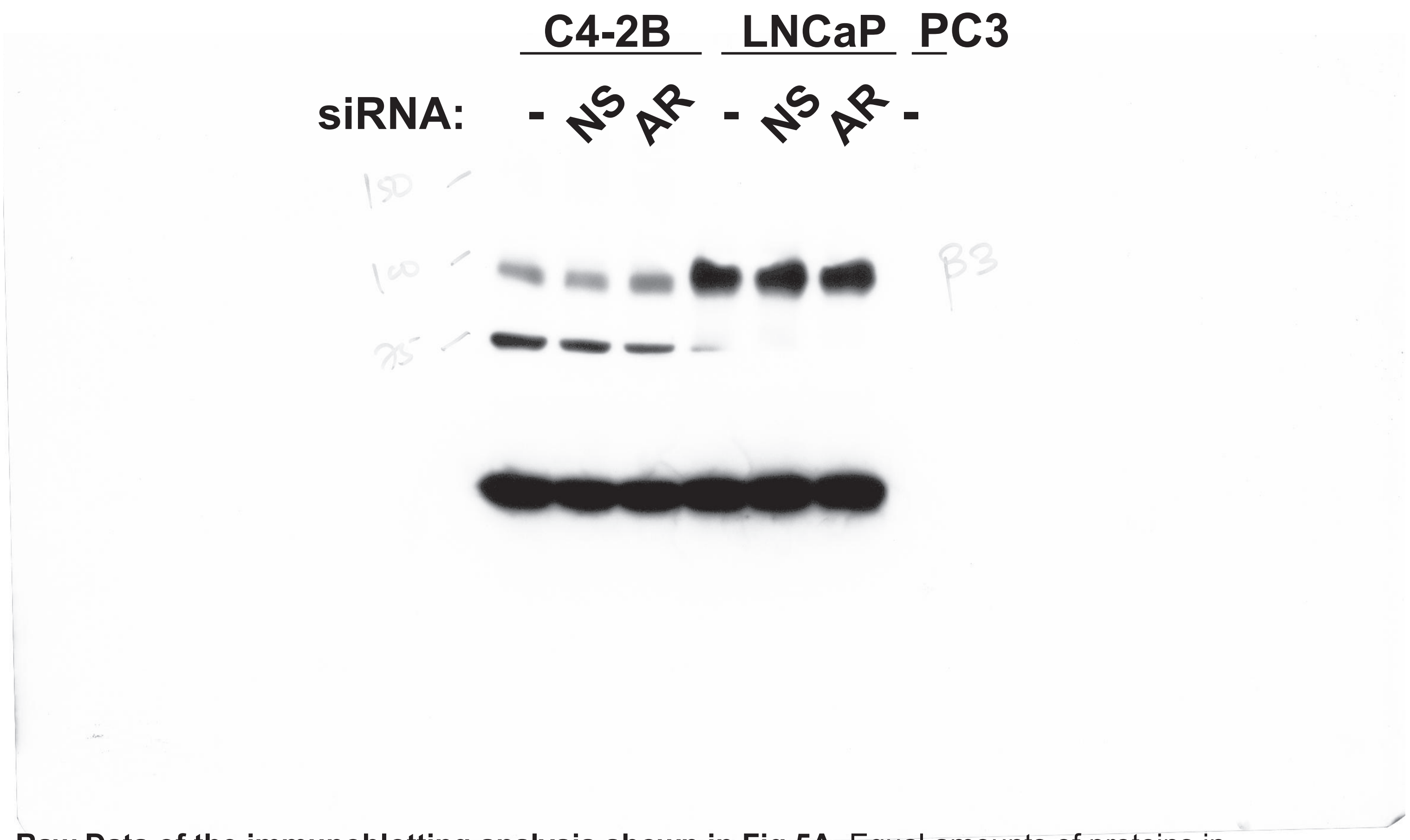

Raw Data of the immunoblotting analysis shown in Fig 5A. Equal amounts of proteins in reducing conditions (heated with 2-mercaptoethanol) were separated by sodium dodecyl sulphate-polyacrylamide gel electrophoresis (SDS-PAGE), transferred to polyvinylidene difluoride (PVDF) membranes (immobilon-E PVDF membrane, pore size 0.45 μm, Millipore), blocked with blocking buffers [5% non-fat dry milk in Tris Buffer Saline with 0.1% Tween 20 (TBST)] for 1 h at room temperature, incubated overnight with primary Abs as described above, followed by TBST washes (4 × 10 min) at room temperature, incubation with horseradish peroxidase (HRP)-conjugated anti-mouse or -rabbit secondary Abs for 1 h at room temperature, followed by TBST washes (4 × 10 min) at room temperature. For visualisation, WesternBrightTM ECL HRP substrate kits (Advansta Inc., CA, USA) were used.

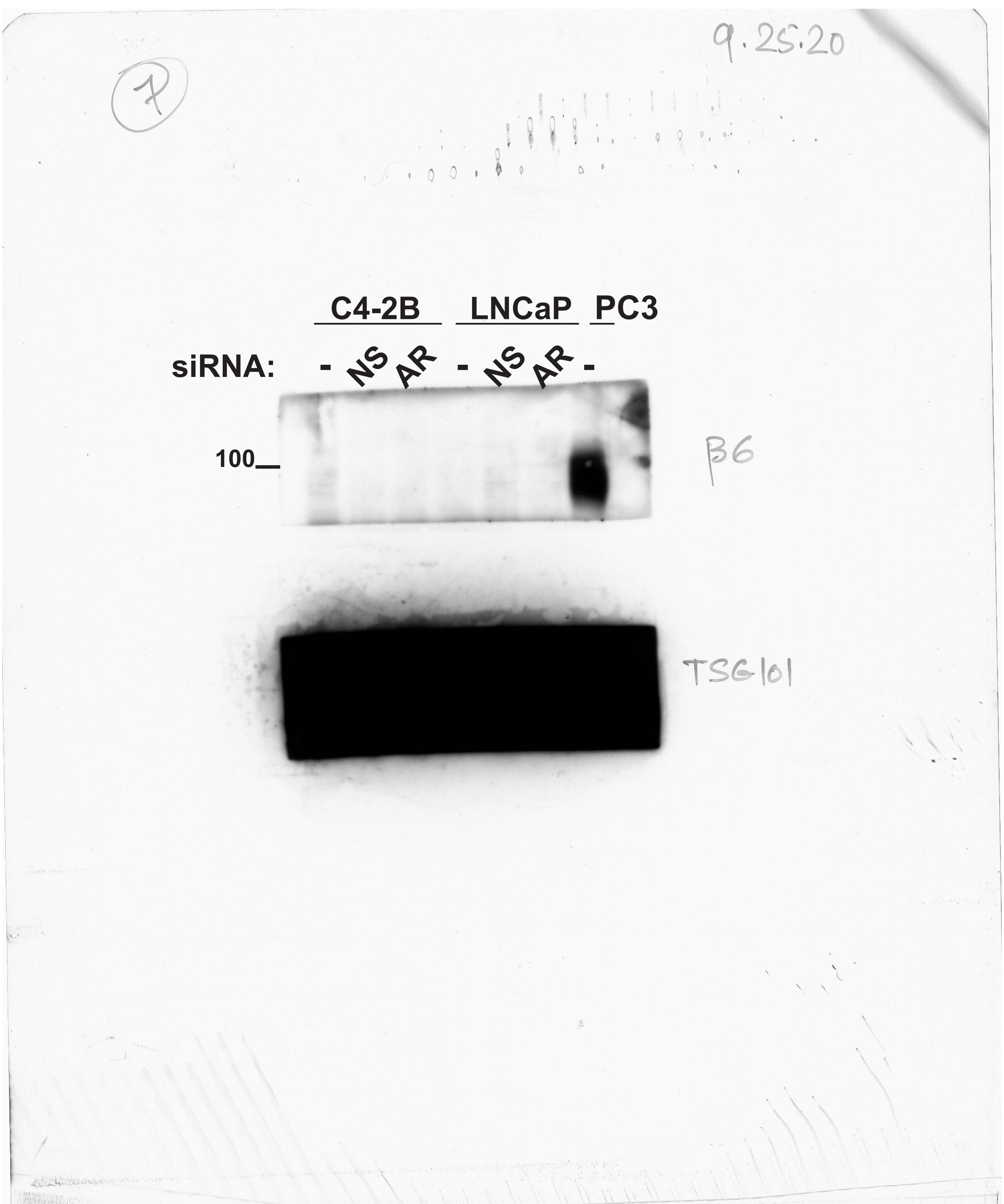

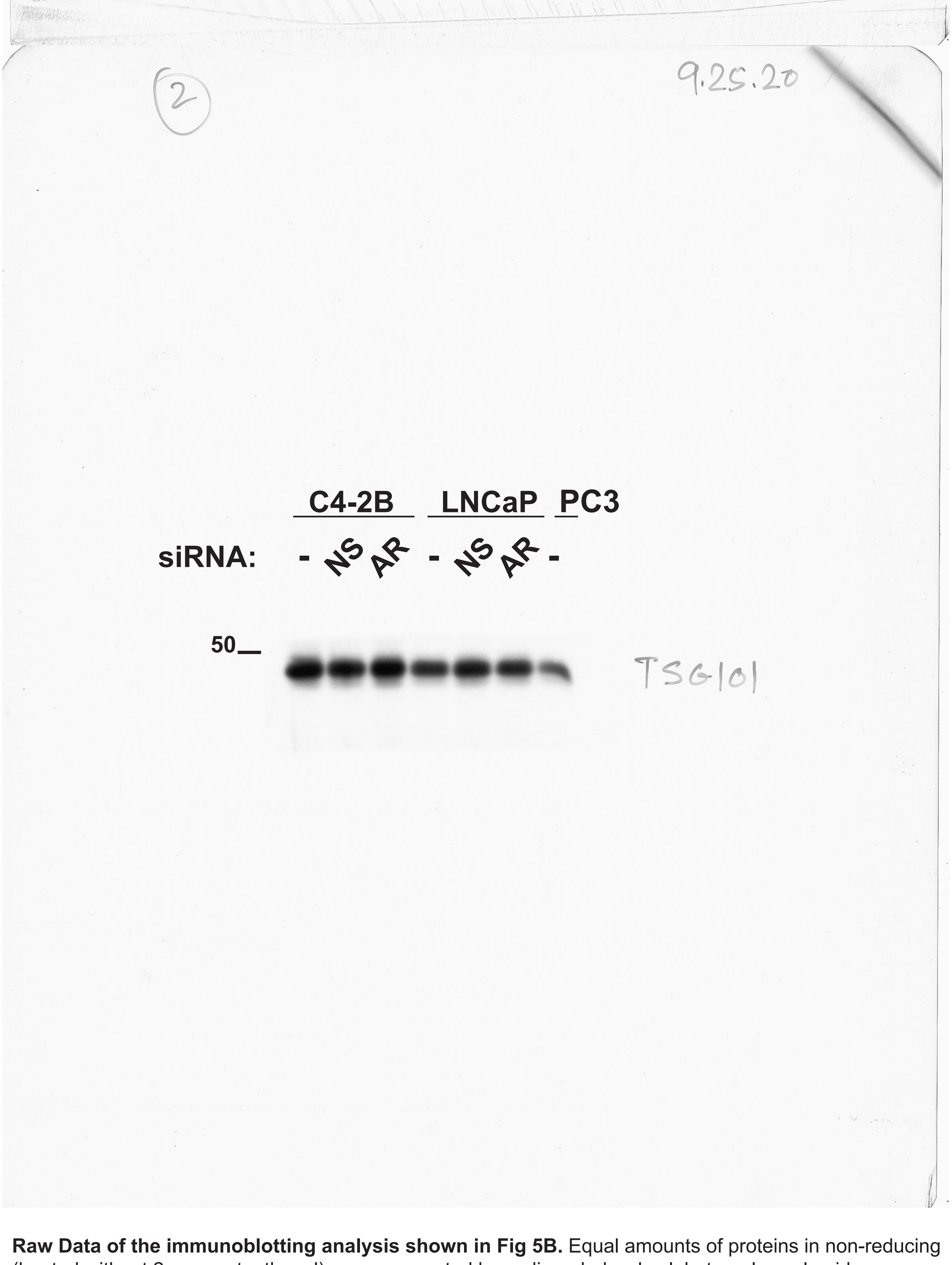

Raw Data of the immunoblotting analysis shown in Fig 5B. Equal amounts of proteins in non-reducing (heated without 2-mercaptoethanol) were separated by sodium dodecyl sulphate-polyacrylamide gel electrophoresis (SDS-PAGE), transferred to polyvinylidene difluoride (PVDF) membranes (immobilon-E PVDF membrane, pore size 0.45 μm, Millipore), blocked with blocking buffers [5% non-fat dry milk in Tris Buffer Saline with 0.1% Tween 20 (TBST)] for 1 h at room temperature, incubated overnight with primary Abs as described above, followed by TBST washes (4 × 10 min) at room temperature, incubation with horseradish peroxidase (HRP)-conjugated anti-mouse or -rabbit secondary Abs for 1 h at room temperature, followed by TBST washes (4 × 10 min) at room temperature. For visualisation, WesternBrightTM ECL HRP substrate kits (Advansta Inc., CA, USA) were used.
